# Emerging Approaches to Profile Accessible Chromatin from Formalin-Fixed Paraffin-Embedded Sections

**DOI:** 10.3390/epigenomes8020020

**Published:** 2024-05-12

**Authors:** Vishnu Udayakumaran Nair Sunitha Kumary, Bryan J. Venters, Karthikeyan Raman, Sagnik Sen, Pierre-Olivier Estève, Martis W. Cowles, Michael-Christopher Keogh, Sriharsa Pradhan

**Affiliations:** 1 EpiCypher Inc., Durham, NC 27709, USA; vishnu@epicypher.com (V.U.N.S.K.); bventers@epicypher.com (B.J.V.); martis@epicypher.com (M.W.C.); 2 Genome Biology Division, New England Biolabs, Ipswich, MA 01983, USA; karthikeyanr@neb.com (K.R.); ssen@neb.com (S.S.); pesteve@neb.com (P.-O.E.)

**Keywords:** chromatin, FFPE, nucleosome, nucleosome-free region, nucleosome-depleted region

## Abstract

Nucleosomes are non-uniformly distributed across eukaryotic genomes, with stretches of ‘open’ chromatin strongly associated with transcriptionally active promoters and enhancers. Understanding chromatin accessibility patterns in normal tissue and how they are altered in pathologies can provide critical insights to development and disease. With the advent of high-throughput sequencing, a variety of strategies have been devised to identify open regions across the genome, including DNase-seq, MNase-seq, FAIRE-seq, ATAC-seq, and NicE-seq. However, the broad application of such methods to FFPE (formalin-fixed paraffin-embedded) tissues has been curtailed by the major technical challenges imposed by highly fixed and often damaged genomic material. Here, we review the most common approaches for mapping open chromatin regions, recent optimizations to overcome the challenges of working with FFPE tissue, and a brief overview of a typical data pipeline with analysis considerations.

## 1. Introduction

**Figure 1:**
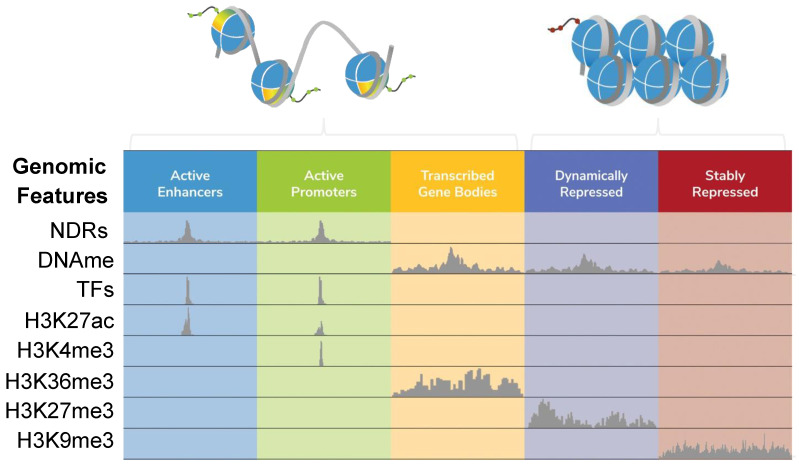
Local features that define ‘open’ and ‘closed’ chromatin. Chromatin states (e.g., active transcriptional enhancers or repressed heterochromatin) can be functionally defined by integrating a range of data elements, including nucleosome-depleted regions (NDRs; mapped by one of the methods discussed in this review), DNA methylation (DNAme (primarily 5-methylcytosine); mapped by bisulfite sequencing or EM-seq [18,19]), transcription factors (TFs) and histone post-translational modifications (PTMs, such as H3K27ac, H3K4me3, H3K36me3, H3K27me3, and H3K9me3) mapped by *ChIP-seq*, or newer approaches like CUT&RUN or CUT&Tag [20,21]. The figure was adapted from [22]. The extended stretch of nucleosome-free DNA at an active promoter represents an NDR.

Alterations in chromatin structure and function are hallmark features of normal cell fate decisions, as well as pathological processes [1,2]. As such, understanding the epigenetic features that regulate chromatin states is essential to develop next-generation biomarkers and therapeutics. The chromatin landscape is defined by the localization of histone post-translational modifications (PTMs), chromatin-associated proteins and RNAs, and the four-dimensional organization of genomic regions [3,4]. Together, these features form a complex molecular language to govern genome transactions [5]. Indeed, gene expression patterns are controlled by the interplay of distinct genomic features (e.g., promoters, enhancers, or heterochromatin) marked by histone PTMs and engaged by chromatin regulatory complexes (e.g., nucleosome remodelers and modifiers) which, in turn, modulate local genome accessibility [5,6,7,8] (Figure 1).

Nucleosomes are the basic repeating unit of chromatin, comprising ~147 bp of DNA wrapped around a histone octamer [9]. ‘Accessible’ or ‘open’ chromatin is conceptually defined as a genomic region containing stretches of free DNA longer than the average linker length between adjacent nucleosomes (~40 bp in human cells) [10,11,12]. These open chromatin regions are commonly referred to as nucleosome-depleted/free regions (NDR/NFR; hereafter NDRs), reflecting dynamic nucleosome turnover and the spectrum of accessibility in population-based assays [13,14,15]. Characterized NDRs contain relatively long free DNA stretches (~120–200 bp), are over-represented in enhancers/promoters, often bound by transcription factors (TFs), and positively correlate with transcriptional activity [16,17].

**Figure 2:**
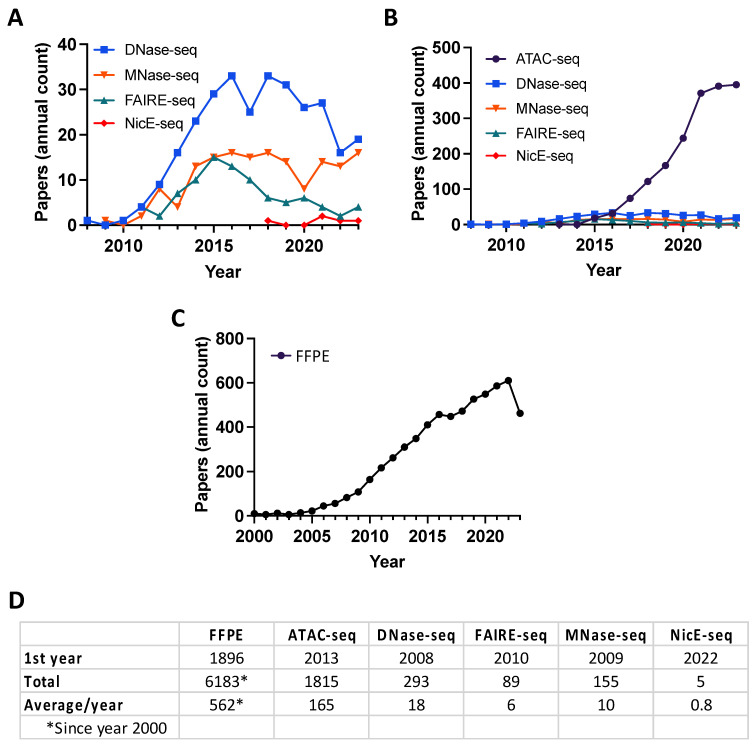
Publication trends. (**A**,**B**) The publication frequency of chromatin accessibility approaches using PubMed search terms “DNase-seq”, “MNase-seq”, “FAIRE-seq”, “NicE-seq”, and “ATAC-seq” (last is the focus of (**B**) to accommodate its overwhelming field adoption rate). (**C**) The publication frequency of PubMed search term “FFPE”. (**D**) Accumulated publications/the first incidence of each search term on PubMed.

A variety of experimental strategies have been developed to map accessible chromatin at the genome scale. Historically, the nuclease DNAse I treatment of chromatin followed by primer extension identified hypersensitive cleavage sites [23,24] representing the NDRs (reviewed in [25,26,27]). Since their commercial release, massively high-throughput sequencing technologies (also known as next-generation sequencing (NGS); in 2005, Roche 454 pyrosequencing; in 2006, Illumina (formerly Solexa) reversible terminators) have revolutionized genome-scale studies by delivering ever-increasing amounts of data at ever-decreasing cost. The first assay to take advantage of NGS for open chromatin mapping was DNase-seq in human CD4^+^ T cells [28], and shortly thereafter, MNase-seq was used to map nucleosome positioning (an indirect approach: see below) in budding yeast [29] (Figure 2A,D). The year 2013 saw the first report of ATAC (Assay for Transposase-Accessible Chromatin)-seq, a Tn5-based assay that was rapidly adopted as the most frequently used chromatin profiling assay (Figure 2B,D).

Whole genome-scale chromatin accessibility assays have delivered breakthrough insights in diverse fields [7,30,31,32]. However, their ability to fully advance clinical research has been hampered by incompatibility with formalin-fixed paraffin-embedded (FFPE) tissue. FFPE is a routine method to preserve clinical samples, with >20 million samples banked each year in the United States alone [33]. This material can be stored for decades at ambient temperatures with minimal degradation of cytoarchitecture and proteomic content [34], making it a potential goldmine for clinical research, especially for rare diseases and longitudinal studies. The first FFPE specimen was reported nearly 130 years ago [35], transforming the face of clinical research and enabling retrospective studies long after initial tissue preservation [36]. Compared to the analysis of native cells, genomic mapping in FFPE tissue presents a number of unique challenges requiring specific protocol modifications and considerations [37]. As an example, sample processing protocols must extract biological material from the paraffin matrix and expose cross-linked chromatin epitopes. However, the primary challenge is genome quality, since FFPE processing induces significant DNA adducts and fragmentation [38]. Further, DNA continues to degrade while in storage, increasing the challenge when analyzing older specimens.

**Figure 3:**
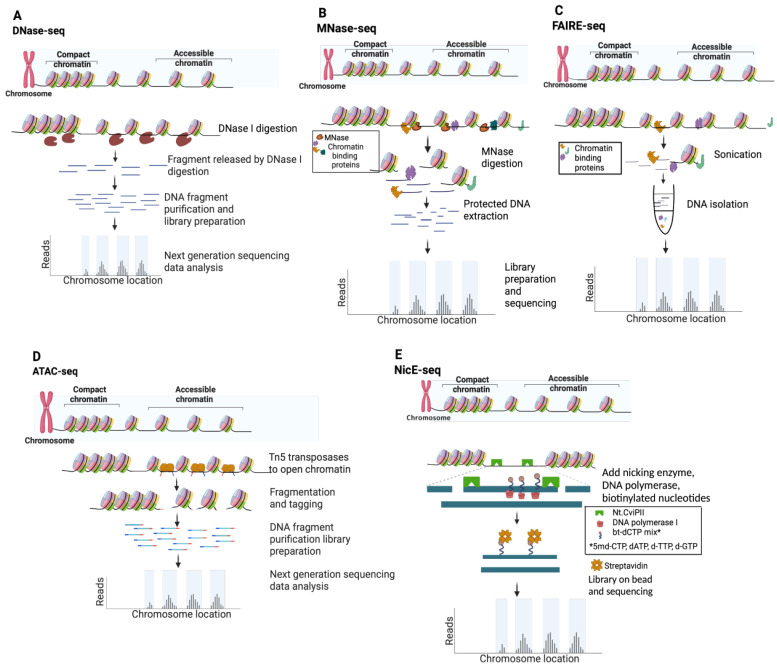
Schematic of techniques used for chromatin accessibility profiling. (**A**) DNase-seq. (**B**) MNase-seq. (**C**) FAIRE-seq. (**D**) ATAC-seq. (**E**) NicE-seq.

**Figure 4:**
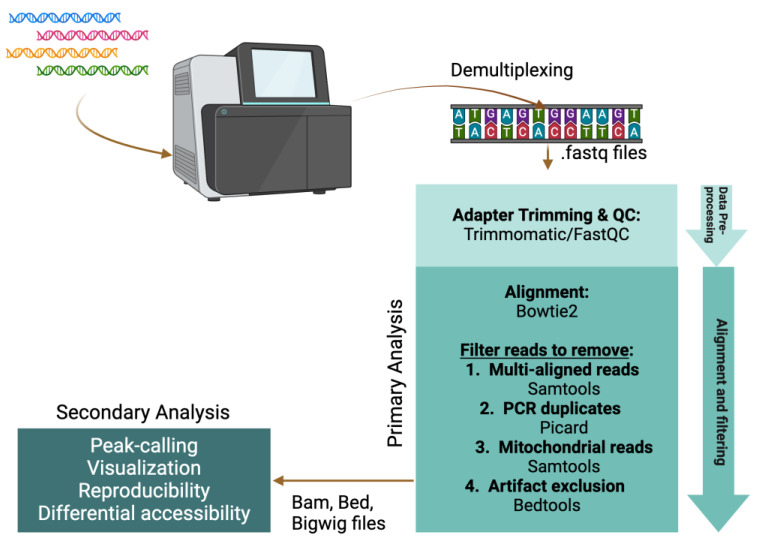
Bioinformatic pipeline for NGS data processing and analysis.

In recent years, there has been increasing interest in chromatin accessibility studies of FFPE tissue, thus mining this potentially rich data seam (Figure 2C,D). The goal of this review is to discuss the most common approaches to map NDRs (Figure 3), their suitability for profiling FFPE samples, and computational strategies to analyze the resulting data (Figure 4).

## 2. Genome-Wide Profiling of Open Chromatin

The most common approaches to map NDRs leverage nucleases, a transposase, a nickase, or the biochemical fractionation of chromatin (Figure 3). For enzyme-based methods, their catalytic properties, molecular size, and potential steric hinderances influence the resulting open chromatin maps.

### 2.1. DNase I Hypersensitivity Mapping Paved the Way for Genome-Wide Open Chromatin Profiling

Deoxyribonuclease I (DNase I) endonuclease (31 kilodaltons, kDa) specifically degrades double- and single-stranded DNA to 5′-phosphate and 3′-hydroxyl [39] and preferentially cleaves accessible chromatin in situ at eponymous DNase I hypersensitive sites (DHSs). In a typical DNase-seq experiment (Figure 3A and Table 1), several million cells are digested to yield DHS subnucleosomal fragments, subsequently identified by library preparation and NGS data analysis [25]. DNase I has proven an excellent tool to study chromatin structure, most notably by the ENCODE consortium [16,27]. In a pioneering study, ~14,000 DHSs were mapped in primary CD4^+^ T cells, and ~90% were shown to be shared across multiple cell types [28]. Although originally thought to lack intrinsic sequence bias, a recent systematic study showed that DNAse I exhibits a C/G preference at the DHS 5′ end [40,41], and several DNase-seq data analysis pipelines have now been corrected for this prejudice [38,39,40,41]. In an effort to map DHSs in FFPE tissue, a more sensitive DNase-seq strategy was developed using a circular carrier DNA-mediated sequencing method (Pico-Seq) [42,43]. However, despite a proof-of-concept study in human follicular thyroid carcinoma, the field has not adopted DNase-seq approaches for FFPE samples (a Pubmed search for “DNAse AND FFPE” only returned two related entries).

### 2.2. Micrococcal Nuclease (MNase) Digestion to Decipher Nucleosome Positioning

*Staphylococcus aureus* MNase has been used to study chromatin for almost five decades [52], and has been employed in the NGS era to map genome-wide nucleosome positioning for multiple eukaryotes (e.g., yeast, worm, fly, mouse, and human) [11,12,13,45,46]. The enzyme is a small (17 kDa), highly processive endo-exonuclease that degrades most types and forms of nucleic acids (e.g., supercoiled, linear, and circular single- and double-stranded DNA and RNA) [39]. These properties enable it to thoroughly digest chromatin until protected by nucleosome structure, cleaving both NDRs and linker DNA. As such, MNase-Seq is distinct from other NDR mapping approaches since it enriches protected DNA (i.e., nucleosome occupancy and position), and open chromatin is then inferred from low-signal regions (Figure 3B and Table 1). MNase shows a strong sequence bias for A/T-rich sequences, and a correction factor is thus built into many data analysis pipelines [44,53]. Recent efforts using MNase to map chromatin accessibility focus on limiting enzymatic digestion [45,54,55], though these titration-based variants have been largely superseded by competing direct NDR mapping methods (Figure 3 and Table 1). Applying MNase-seq to FFPE tissue sections has yielded minimal success, with a Pubmed search for “MNase AND FFPE” returning zero entries.

### 2.3. FAIRE-Seq Identifies Accessible Chromatin Regions through Principles of Biochemical Separation and Solubility

FAIRE (Formaldehyde-Assisted Isolation of Regulatory Elements) identifies NDRs by building on the observation that transcriptionally active chromatin shows differential biochemical solubility after formaldehyde fixation [56]. In brief, cells are treated to cross-link chromatin, sheared by sonication, and undergo phenol–chloroform extraction, where the aqueous phase contains DNA fragments associated with NDRs (Figure 3B) [57]. While FAIRE-seq does not have the cleavage bias of sequence-specific nucleases [40,44], it is highly dependent on cross-linking efficiency and often undermined by a poor signal-to-noise ratio [57,58], false positives [46], and the challenge posed by low cell numbers [59] (Table 1). Nevertheless, FAIRE-seq has been widely applied to model systems and cell lines [57,58,59,60,61], particularly as a part of ENCODE efforts to systemically identify active regulatory elements [16]. A recent report mapping open chromatin by FAIRE-seq cleverly circumvented the challenge *Drosophila* pupa cuticles present to in situ enzyme-based methods, thus providing higher quality data than ATAC-seq [62]. Despite this, over the past decade, ATAC-seq has clearly emerged as the preferred assay to map chromatin accessibility, while FAIRE has declined in use (Figure 2A,B).

### 2.4. Tn5 Transposon Tagmentation of Accessible Chromatin (ATAC-Seq)

Tn5 transposase was first discovered in the 1970s based on the kanamycin resistance it conferred to host bacteria [63,64]. In addition to providing a mechanistic model for transposases, Tn5 (106 kDa active dimer) has become an invaluable molecular tool [65]. Most recently, it has been leveraged to identify NDRs via ATAC-seq and to map histone PTMs via CUT&Tag [20,66]. ATAC-seq is currently the most widely used open chromatin mapping assay (Figure 2B) due to its relative speed, efficiency, and sensitivity (Table 1). The approach employs a genetically engineered hyperactive Tn5 transposase to insert loaded DNA adapters preferentially at accessible DNA in situ (i.e., tagmentation) for direct PCR amplification and NGS [47,66] (Figure 3D). Tn5 displays an enzymatic sequence bias which, while more complex than that of the nucleases used for NDR mapping, can also be compensated at data analysis [53,67]. With deep enough sequencing, TF binding footprints may also be inferred from protected fragments within the NDRs [48,68]. Early versions of the ATAC-seq protocol were hampered by high read duplications and contaminating mitochondrial DNA, which, together, consumed a majority of sequencing bandwidth. These issues were largely circumvented by the development of Omni-ATAC wherein nuclei were isolated with a cocktail of detergents to remove contaminating mitochondria, increasing the library complexity and signal-to-noise ratio [69,70].

Beyond the application of ATAC to interrogate model organism and cell line epigenomes, recent efforts have explored if the enzyme could be applied to clinical studies on FFPE tissue sections [71,72,73,74,75]. Obtaining PCR amplicons from Tn5-based approaches requires two independent tagmentation events in opposing orientation and in close proximity (<~700 bp). This inherently reduces library complexity and effective yields and is exacerbated by the highly damaged DNA in FFPE material. To address this, a recent approach followed Tn5 tagmentation with an in vitro transcription (IVT) step, such that a single insertion event could be amplified by T7 RNA polymerase [72,73,74,75]. While standard ATAC-seq yielded some success in nuclei isolated from mouse FFPE liver and kidney, the Tn5-IVT-modified approach improved the library complexity, signal-to-noise ratio, and other key metrics. However, this approach is limited by a lengthy and complex procedure that relies on harsh chemical, mechanical, and enzymatic methods (e.g., xylene, needle shearing, and a collagenase/hyaluronidase cocktail) to extract nuclei from FFPE tissue. Further, such exacting handling contributes to genome fragmentation [76,77].

Noting these observations, Henikoff and colleagues instead used gentle heating and permeabilization, similar to how FFPE sections are routinely deparaffinized for histological analysis, to prepare samples for CUT&Tag [71]. NDRs facilitate access to DNA by the transcriptional machinery, so the CUTAC (Cleavage Under Targeted Accessible Chromatin) protocol was developed to target Tn5 to active chromatin via RNA Pol II, and yield short tagmentation fragments (~60 bp) to reduce the impact of DNA damaged samples. Of note, FFPE-CUTAC yielded higher quality data than FFPE-ATAC from mouse brain [71], suggesting the potential of innovative Tn5-based approaches to map the epigenome of clinically relevant FFPE samples. In a subsequent study to understand hyper-transcription in cancer, FFPE-CUTAC was successfully applied to mouse and human nuclei isolated from FFPE scrolls or in situ from FFPE tissue sections [78]. While these recently emerging approaches appear to hold great potential, it remains to be seen how widely they will be adopted.

### 2.5. Nicking Enzyme-Assisted Accessible Chromatin Sequencing (NicE-Seq)

NicE-seq is a recent approach (Figure 2A,B) that excels at NDR profiling from heavily fixed cells, including FFPE [50,51,79,80]. In contrast to nuclease or Tn5 transposase double-strand cleavage (as above), *Chlorella* virus Nt.CviPII (63 kDa) is a nicking endonuclease that cuts only one strand of dsDNA at CCD sites (D = A/G/T), which occur by chance every ~21 bases [49]. In the latest version of the protocol (One-pot UniNicE-seq), Nt.CviPII nicks at NDRs are filled in using an NTP mix containing biotinylated- and 5-methyl-dCTP triphosphates (to, respectively, label and prevent further nicking), the genome is enzymatically sheared, biotin-labeled DNA is captured on streptavidin beads, and libraries are prepared on the matrix by PCR (Figure 3E). This method is a fast, simple, and robust one-tube workflow, although it is incompatible with native cells and yields larger DNA fragment sizes than ATAC-seq, which limits resolution. NicE-seq has now been applied to a wide variety of mouse and human cell lines, primary tissues, and FFPE sections [50,80]. The reason why NicE-seq prefers heavily cross-linked samples over native samples is not entirely clear, but may be due to nicking activity outside NDRs; in this regard, filling nicks with nucleotides to label CCD sites and restrict further cleavage events provided dramatic signal improvements as the protocol evolved [50,51,79,80]. Central to the theme of this review, NicE-seq identified NDRs in human lung and liver FFPE samples from as few as five thousand cells (Table 1) [50,80]. Similar to FFPE-CUTAC, NicE-seq can be performed on permeabilized and minimally disrupted FFPE sections in situ, obviating the need for nuclei purification by harsh methods that damage genomic DNA. As such, the approach shows great potential for broad adoption to map open chromatin in clinical FFPE material.

## 3. Data Analysis

Several excellent papers describe methods to analyze data from ATAC-seq [81,82,83], the most popular open chromatin profiling approach (Figure 2). Instead, this review aims to provide a brief overview of the key considerations and data analysis pipelines that are broadly applicable to ATAC-seq, FAIRE-seq, and NicE-seq (Figure 4). Because of their distinct data structures, DNase-seq and MNase-seq each require specific pipelines [81]. For example, open chromatin from MNase-seq data is inferred from nucleosome-centric maps, and central considerations are made to identify the nucleosome dyad, account for MNase sequence cleavage bias, and quantify nucleosome occupancy and ‘fuzziness’ [29,45,55].

The major features of a typical ATAC-seq pipeline involve: (1) read pre-processing and quality control (QC); (2) primary analysis (read alignment and filtering); and (3) secondary analysis (peak-calling, visualization, reproducibility, and differential accessibility). Paired end (PE) sequencing is highly recommended as this informs on DNA fragment length: an important metric for assay success and interpretation. A sequencing depth of 30–50 M PE reads is usually sufficient for good genome coverage, but this will depend on how much bandwidth is consumed by mitochondrial DNA contamination and read duplicates.

### 3.1. Read Pre-Processing and Quality Control

Prior to alignment, several tools are used to assess the quality of the library and sequencing run. FastQC reports on base calling quality and overrepresented sequences, such as primer and adapter dimers. Low base calling scores (<20 Q-score) often indicate a poor-quality library and/or sequencing run. Overrepresented primer and adapter dimers are not as problematic in Tn5-based libraries as in ligation-mediated PCR libraries. If the accessible DNA fragment length is not greater than twice the paired-end read length, sequencing will read through to the Illumina adapter regions. Such readthrough can negatively impact genome alignment, so read trimming tools (e.g., Trimmomatic [84]) are used to detect and prune Illumina adapter sequences.

### 3.2. Primary Analysis Pipeline

Genome alignment is typically the most time-consuming and computationally intensive step in the primary pipeline, spurring the development of fast, memory-efficient aligners optimized for short paired-end reads (e.g., Bowtie2 [85]). The goal is to identify the unique genomic location that corresponds to each read pair. However, multi-aligned reads pairs are common and must be flagged/removed from further analysis since they introduce ambiguity. In addition to removing multi-aligned reads using Samtools [86], other utilities (e.g., Bedtools [87,88] and Picard (http://broadinstitute.github.io/picard [accessed 1 May 2024])) are used for read processing and filtering to remove PCR duplicates, contaminating mitochondrial DNA, and artifactual exclusion list regions [89].

### 3.3. Tools for Secondary Analysis

Identifying and visualizing statistically enriched NDRs enables data interpretation and provides biological insights. Accessible chromatin occurs in relatively narrow regions that can be identified with peak-calling tools, such as MACS2 [90]. DeepTools2 [91] is an excellent suite of utilities to assess data reproducibility, generate signal heatmaps, and process alignment files for visualization in genome browsers, such as Integrative Genomics Viewer [92]. EdgeR (originally developed for RNA-seq analysis) is widely used to identify NDR peak locations that display a statistically significant differential signal across two conditions (e.g., pre or post drug treatment) [93]. Additional tools can provide further insights to open chromatin patterns but are outside the scope of this paper, and we recommend one of the more comprehensive data analysis reviews [81,82,83].

## 4. Discussion

Identifying NDRs throughout the genome provides a window into transcriptionally active regions in normal and disease states. Chromatin accessibility profiling has enormously impacted basic and pre-clinical research and is of extreme interest for application to clinical biopsy specimens in FFPE blocks. The goal of any useful genomics method is to yield the maximum amount of high-quality DNA (or RNA) to QC defined metrics. However, major challenges associated with FFPE tissues generally slow the application of epigenomic approaches to archived biopsy specimens.

There are three main areas of focus if users are to generate high-quality data from FFPE tissues: (1) best practices during clinical tissue preparation and preservation; (2) improved methods of material preparation; and (3) the optimization of genomic assays specifically for FFPE material. The first is largely outside end-user control, though minimizing any delay between tissue harvesting and fixation and shorter storage times improve DNA integrity and assay yields [94]. Assessing genome integrity by DNA fragmentation or PCR analyses can inform sample quality and suitability for chromatin accessibility profiling [95]. Tissues are generally processed by nuclei extraction or in situ permeabilization [71,73], and improved methods should balance increased yields without further compromising the genomic integrity. FFPE repair kits are available [96], but their impact on data quality for open chromatin profiling remains to be determined. Finally, lessons may be learned from efforts to develop RNA-seq and ChIP-seq for archived FFPE material [76,94,97]. For example, the cross-linking reversal step is a major source of DNA fragmentation [94,96], but this can be mitigated by high concentrations of Tris, which improved yields by three-fold and resulted in longer DNA fragments [77].

Because bulk genomic studies represent cell populations, heterogeneity is a potential caveat in data interpretation. This is particularly the case when using FFPE primary tissue samples derived from patient biopsies. Single cell genomic methods have helped to reduce the heterogeneity blind spot [98], though FFPE tissues continue to provide a challenge for such approaches. However, spatial transcriptomic and epigenomic approaches with FFPE sections are emerging, which can help to assess heterogeneity and provide valuable context for orthogonal studies [99,100,101,102].

It is a given that the direct analysis of primary tissue provides insights to the development of human disease. Indeed, a histopathological analysis of FFPE brain samples has been central to characterize mechanisms of normal and pathological aging [103,104]. The ability to perform comprehensive epigenomic analyses in such samples could provide further understanding of these processes and revolutionize clinical research.

## Figures and Tables

**Table 1:** Commonly used approaches for chromatin accessibility profiling.

	DNase-Seq	MNase-Seq	FAIRE-Seq	ATAC-Seq	NicE-Seq
**Type of input cells/tissue**	Fresh/formaldehyde cross-linked/FFPE (Formalin-Fixed Paraffin-Embedded)	Fresh/formaldehyde cross-linked	Formaldehyde cross-linked	Fresh/formaldehyde cross-linked (less efficient in fixed)	Formaldehyde cross-linked/FFPE
**Application to FFPE (PubMed)**	1	0	1	2	2
**Number of input cells**	10^6^–10^7^	10^3^–10^7^	10^3^–10^7^	1 cell—5 × 10^4^	25 cells—10^5^
**Fragment size (i.e., resolution)**	~200 bp	~200 bp	~300 bp	~100–200 bp	~300 bp
**Key features**	DNase I (endonuclease) cuts unprotected DNA	MNase (endo-exonuclease) digests unprotected DNA	Sonicate unprotected DNA in cross-linked material	Tn5 transposase tagments open region with DNA adapters	Nt-CviPII nickase cuts/labels CCD sites in unprotected DNA
**Sequencing type**	Single/paired end	Single/paired end	Single/paired end	Single/paired end	Single/paired end
**Target region**	NDR	Linker DNA between Nucleosomes	NDR	NDR	NDR
**Sequencing depth (human genome; ~3 billion bp)**	20–50 million mapped reads	150–200 million mapped reads	20–50 million mapped reads	25–30 million mapped reads (non-mitochondrial)	20–30 million mapped reads
**Cleavage bias**	Yes	Yes	No	Yes	Yes
**Advantages** **/** **disadvantages**	No prior knowledge of the sequence or binding protein is required **/** time consuming, requires laborious enzyme titrations and calibrations, requires high sequencing depth	Nucleosome positioning can be inferred **/** requires laborious enzyme titrations and calibrations, requires high sequencing depth, indirect profiling of open regions	No enzymes optimization or titration required **/** low signal-to-noise ratio, relatively complex computational data analysis and interpretation, results are highly fixation-dependent	Simple, fast, and sensitive approach; high signal-to-noise ratio **/** High mitochondrial DNA counts (unless nuclei isolated), requires two independent tagmentation events in opposite orientation, Tn5 sequence bias and promoter-enrichment bias	Simple enzymatic approach, <5% mitochondrial DNA counts, optimal in fixed or FFPE samples, can be used in clinical settings, efficiently profiles promoters and enhancers **/** AT-rich sequences may be underrepresented
**References**	[26,28]	[12,13,14,44,45]	[46]	[47,48]	[49,50,51]

## Data Availability

Not relevant.

## Abbreviations

ATAC-seq, assay for transposase-accessible chromatin using sequencing; DHS, DNAse I hypersensitive site; DNAme, DNA methylation; ENCODE, encyclopedia of DNA elements; FAIRE, formaldehyde-assisted isolation of regulatory elements; FFPE, formalin-fixed paraffin-embedded; NDR, nucleosome-depleted region; NFR, nucleosome-free region; NGS, next-generation sequencing; PTM, post-translational modification; TF, transcription factor.
